# The Experiences of COVID-19 Patients in Intensive Care Units: A Qualitative Study

**DOI:** 10.1177/00302228211024120

**Published:** 2021-06-12

**Authors:** Meltem Kürtüncü, Aylin Kurt, Nurten Arslan

**Affiliations:** 1 Nursing Department, Faculty of Health Sciences, Zonguldak Bülent Ecevit University, Turkey; 2 Nursing Department, Faculty of Health Sciences, Bartın University, Turkey; 3 Nursing Department, Zonguldak Bülent Ecevit University, Turkey

**Keywords:** COVID-19, patients, intensive care units, qualitative research, pandemics

## Abstract

This exploratory qualitative study explores the experiences of COVID-19 patients in intensive care units and after discharge. Semi- structured telephone interviews were conducted with 18 COVID-19 patients admitted to and discharged from intensive care units between March and September in 2020. The themes of this study were determined as “feelings about the illness and intensive care,” “psychological and physical damages,” “nurses’ efforts and the importance of care.”, and “protecting health and life”. COVID-19 patients in intensive care units may experience permanent physical and psychological damages. The findings suggest that the first step in carrying out interventions in the intensive care units is to ensure that continuous communication with patients is maintained so that their orientation to the new circumstances can be achieved. Nursing interventions to patients missing their families can have compensated for the loss of family support and care during their critical illness.

Coronavirus disease 2019 (COVID-19) is a virus causing severe pneumonia that emerged in Wuhan, China and then quickly spread out all over the world (Huang et al., 2020; She et al., 2020). Discovered close to the end of 2019, COVID-19 was proclaimed a pandemic by the World Health Organization as of March 11, 2020 when it was seen to affect a total of 118.000 people in 114 countries, causing the death of 4.291 (World Health Organization, 2020b).

The morbidity and mortality rates of COVID-19 are largely related to Acute Respiratory Distress Syndrome (ARDS) caused by acute viral pneumonia. Some patients with the disease commonly experience difficulty in breathing and hypoxemia (Millar, 2020; Xu et al., 2020). Among severe cases, 10%-20% display developed symptoms of ARDS between day 8–14 and may need mechanical ventilation due to noncardiogenic pulmonary edema. Patients with a critical shock profile and respiratory distress serious enough to necessitate mechanical ventilation may need to be admitted into the intensive care unit (Rothan & Byrareddy, 2020; Xu et al., 2020).

The time in the intensive care unit under normal circumstances may be quite challenging for patients. In the COVID-19 pandemic, the number of admissions to intensive care units (ICU), especially at the beginning, increased rapidly (Arabi et al., 2020). Upon admission, patients were intubated and sedated (Wang et al., 2020). The fast pace of the progression of disease may make it hard for patients to adapt to their condition. Patients who do not lose consciousness can be lead into depressive thoughts as they watch other patients being quickly intubated and sedated, wondering whether they would be experiencing the same fate (Righy et al., 2019). The physical appearance of the healthcare team, clad in their protective equipment during treatment and care procedures is another cause of anxiety for patients. Additionally, the prohibition of visitors in the ICU due to the pandemic is another psychological factor that is particularly distressing for patients (Millar, 2020). It is also noted that because of the long length of time COVID-19 patients must spend under intensive care, connected to mechanical ventilators and being sedated, the return of these individuals to their previous routines after enduring treatment for such a serious disease may be a huge challenge in and of itself (Sun et al., 2020). As a result, patients may show signs and symptoms of post-traumatic stress syndrome after discharge (Righy et al., 2019). The circumstances of intensive care in the time of the COVID-19 pandemic is in short much more challenging than usual (Wang et al., 2020; Yang et al., 2020).

The severity of intensive care conditions implies that patients can be affected with severe outcomes (Righy et al., 2019). There are qualitative studies in the literature about the challenges healthcare personnel are facing during the COVID-19 pandemic (Liu et al., 2020; Sun et al., 2020). One of these qualitative studies examines the psychological experiences of patients hospitalized for COVID-19 (Sun et al., 2020). No study was noted however that examined the experiences of patients during their treatment in the ICU for COVID-19 nor after their discharge from the unit. We aimed to review the experiences of COVID-19 patients while under intensive care and afterwards. In the context of this general aim, our study questions were the following: (1) What kind of experiences/feelings did the patients in ICU? (2) How did the patients continue with their lives after their discharge from the hospital? (3) How did having COVID-19 and being treated in the intensive care unit affect the patients? (4) What do the patients experience in a pandemic different from hospitalization and discharge in intensive care compared to the pandemic before the pandemic?

## Methods

### Study Design and Participants

An exploratory qualitative study was performed with a purosive sample. In-depth, semi- structured individual interviews were conducted, transcribed verbatim and submitted to thematic analysis by three independent researchers.

By using a purposeful sampling method, we selected 18 patients with COVID-19 in Zonguldak Atatürk State Hospital between March and September in 2020. The inclusion criteria included: (1) Being a COVID-19 patient in an intensive care unit and discharged (not lost), (2) Not having a speech disability (3) Agreeing to participate in the study voluntarily. We determined the number of required respondents by interviewing patients who met the inclusion criteria until the data were saturated and no new topics were generated. The participants’ characteristics are shown in Table 1.

**Table 1.** Participants’ Characteristics.

Participants	Gender	Age	Chronic illness	Time in intensive care (days)
P1	Male	59	None	7
P2	Male	67	Cerebrovascular Event	9
P3	Male	68	Hypertension, Chronic Heart Failure, Cerebrovascular Event	35
P4	Male	39	Generalized Anxiety Disorder	2
P5	Male	64	Hypertension, Chronic Heart Failure, Diabetes Mellitus	4
P6	Male	48	Diabetes Mellitus	13
P7	Male	55	Hypertension	20
P8	Male	77	Hypertension, Ischemic Heart Disease, Prostate Hyperplasia	14
P9	Male	48	Hypertension	8
P10	Female	60	Diabetes Mellitus	22
P11	Male	71	None	12
P12	Male	67	Hypertension, Diabetes Mellitus	4
P13	Male	49	Diabetic Polyneuropathy	39
P14	Female	62	Hypertension, Diabetes Mellitus, Ischemic Heart Disease, Chronic Kidney Failure	14
P15	Male	70	Hypertension, Chronic Kidney Failure	10
P16	Male	64	Hypertension	34
P17	Female	64	Peripheral Vascular Disease, Diabetes Mellitus	4
P18	Female	75	Cerebrovascular event	3

### Data Collection

Due to isolation and the preventive measures taken, data were collected by telephone. The information and telephone numbers of the patients was obtained with written permission from the hospital administration. The three authors (MK, AK, NA) contacted the patients prior to the interview to ascertain if there were any changes in their condition. The participants were informed about the identity of the researchers. They would be talking to and given information about the study (its purpose, the confidentiality of the responses, where and how the data would be kept). Participation was on a volunteer basis. The researchers who have a master's and doctoral degree in nursing and had some studies in the field of qualitative research. The researchers knew and had applied semi-structured in-depth interview techniques many times. Also, there was no relationship between the researchers and the participants. The researchers did not know the patients. In this way, we prevented interviewer bias and reflexivity (Palaganas et al., 2017). The participants were asked for their consent to have the discussions recorded on a voice recorder. The interview sessions each took between 30–45 minutes.

Data were collected with a “Semi-structured interview form” that was prepared by the researchers based on the literature (Liu et al., 2020; Sun et al., 2020). The Interview Form contained open-ended questions regarding the experiences of the COVID-19 patients in ICU. The questions in this form were: (1) What were your experiences during the time you were in intensive care? (2) How and what did you feel when you were in intensive care? (3) How has your life been since you were discharged from the hospital? (4) How did COVID-19 affect you? (5) What do you think about the outcome of your sickness?

### Data Analysis

Data analysis was independently performed by three authors (MK, AK, NA) and occurred in three phases: data reduction; data display; and conclusion drawing/verification (Miles & Huberman, 1994). For data reduction, significant segments of the interview were coded into themes. All interviews were carefully read to get a comprehensive picture, and interpretative notes were made. Emerging subthemes were subsequently grouped into major themes. The data display permitted drawing conclusions. Maps of themes and quotations were organised to facilitate data analysis. Conclusion drawing/verification implied that the researchers reviewed the meaning of the analysed data, confirming emergent conclusions as a means of testing the validity of the findings (Miles & Huberman, 1994). Themes and sub-themes were reviewed and iterated to guarantee that they reflected the data collected. No substantial differences were found between the two researchers. The research team conducted frequent online group meetings to discuss topics and ensure reflexivity. Discrepancies were solved by discussions and coming to consensus with experienced peers within the research team. Patients were contacted if doubts arose during data confirmation. Feedback regarding the rigour and trustworthiness of the themes was also gathered from the patients who had been interviewed (Lietz et al., 2006). The study was presented in accordance with Consolidated criteria for reporting qualitative research (COREQ) checklist for qualitative research (Tong et al., 2007).

### Ethical Considerations

Ethics committee approval for the study was obtained from ethics committee (Date: 10/14/2020 and Approval No. 2020/20) and from the administration of the institution where the study would be conducted. Data collection was performed based on the voluntary participation. The patients were informed about the aim of the study, the confidentiality of all data. All participants signed informed consent in digital form. In compliance with the requirements of research ethics, the names of the patients were not used but assigned code names.

## Results

The themes emerging from the patient interviews, as ascertained by Colaizzi’s analysis, can be seen in Table 2.

**Table 2.** Themes and Subthemes.

Themes	Feelings about the illness and intensive care	Psychological and physical damages	The dedicated work of the nurses and the importance of care	Protecting health and life
Subthemes	Fear (n = 16)	Psychological damage (n = 18)	Selfless care (n = 15)	Heeding the rules of isolation and hygiene (n = 13)
	Denial (n = 7)	Sustaining physical damage (n = 4)	Communication (n = 10)	Paying attention to nutrition (n = 8)
	Loneliness (n = 8)	Problems with adapting to daily life (n = 9)		Psychological maturity (n = 9)

### Theme 1: Feelings About the Illness and Intensive Care

Sixteen of the participants said they were very afraid to be admitted into intensive care and felt that they would not get out alive.

There was a patient beside me, he was living, he was looking at me. Then, 5-6 minutes later I saw he’d been put into a body bag. I thought to myself, the same thing can happen to me, I’m going to die. It was terrible. (P1)

I don’t know how I got there. I was unconscious. When I woke up, I said to myself, ‘Where am I? These people are wearing very strange outfits.’ I just didn’t know what to do. (P10)

Some of the patients feared for their life because of the fact that COVID-19 was unknown territory.

I felt the aura of death. It’s an unknown disease, there’s no cure. I had prepared myself for everything. (P6)

When they said the word ‘corona,’ I was filled with fear. I was following it up on TV. Everybody was dying. I thought, I’m going to die too. (P16).

Seven of the patients revealed that they denied that they had COVID-19, that they couldn’t accept it. Some of them did not want to go into the hospital. Some of them said:

I went to the emergency room with palpitations. You have COVID-19, they said. I couldn’t believe it. They wanted to put me in intensive care. I refused. They said if I don’t consent to being admitted, I’d be committing a crime. (P4)

I’m an athlete. I walk, I swim. I eat a lot of honey. I was very healthy. I don’t know how this disease found me. I still can’t accept it. (P11).

Eight of the patients said they felt a lot of fear during the time they were in intensive care, revealing that the one thing that made it hardest to cope was the feeling of being alone. Some of the patients expressed themselves in this way:

I couldn’t see my loved ones for a long time. I felt completely alone. One of the healthcare workers called my son on the telephone. I thought the world had been handed to me. (P17)

I couldn’t see anyone in my family. I was going mad. (P11)

### Theme 2: Psychological and Physical Damage

All of the patients reported that their having to be in the intensive care unit and the uncertain duration of their illness was psychologically detrimental. Some of the patients had this to say:

People are lying there naked like corpses. When anyone mentions ‘intensive care,’ I simply rebel. (P5)

If I didn’t have much faith, I would definitely have lost my mind in there. (P15)

Four patients said that they sustained physical damages after recovering from the illness. Some of the patients expressed themselves by saying:

My lungs developed 16-17 lesions from the disease and from the stress. Now I need to cope with this. (P12)

When I was released, they found a stone in my bladder. I’m being treated for this these past few months. (P8)

Half of the patients said they had difficulty adapting to their daily life routines because of the psychological and physical damages. Some of the patients reported:

I was healthy when they admitted me into intensive care; I came out sick. My friends at work all asked, ‘What’s happened to you?’ I’m about to be fired from my job. I’m a mess psychologically. I’ve started therapy. (P4)

It’s been four months since my discharge. But I still can’t go back to work. I can’t even go out. (P6)

### Theme 3: The Dedicated Work of the Nurses and the Importance of Care

Fifteen of the patients said the nurses were extraordinarily dedicated and that they felt they were beholden to them for their efforts. Some of the patients had this to say:

I never knew how nurses worked. I saw this when I was in intensive care. They put their own lives at risk saving our lives. (P16)

I feel like crying every time I think about the nurses working there. They saved my life. (P13)

Communication was one of the most important matters for the patients. They said that the nurses boosted their morale and supported them in reducing their feelings of fear and loneliness.

The nurses took very good care of me. I couldn’t sleep at night. We would chat together up until the morning. They acted like family. Each of them is an angel. (P14)

They came to my bedside every day. They asked me how I was feeling. They drew the curtains between me and the neighboring patient so I wouldn’t be afraid. (P13)

### Theme 4: Protecting Health and Life

All of the patients said they had learned to comply with the rules of isolation, hygiene, and nutrition.

I never go out without a mask anymore. I’m careful about social distancing. I don’t go into crowds. I don’t want to go back into intensive care. (P9)

I lost a lot of weight with this illness. I couldn’t eat, I couldn’t drink. I was too tired to even walk. I’m very careful now with what I eat and drink. That’s the only way I can get better. (P18)

Nine patients underlined the fact that they held on closer to life and realized the importance of good health. Some of the patients’ statements were the following:

When I got back home, I felt like kissing even my house plants. I realized the value of life. (P2)

We don’t think about it but it turns out that being able to breathe is a valuable thing. (P17)

I felt like a new-born child when I got well. I feel like I’ve been given a second chance to live. (P3)

## Discussion

The present study explored the psychological experiences of COVID-19 patients in the intensive care unit using phenomenological methods. The themes were: (1) feelings about the illness and the intensive care unit; (2) the dedicated work of the nurses and the importance of care; (3) psychological and physical damage; and (4) protecting health and life.

The theme of “feelings about the illness and the intensive care unit” was derived from the testimonies of the COVID-19 patients revealing their psychological symptoms such as fear, denial and loneliness and their experiences with the illness in the intensive care unit. Feelings of fear are rampant among patients due to the high mortality rates associated with the COVID-19 pandemic (Luo et al., 2020; Sun et al., 2020). COVID-19 patients experience a concentration of feelings of fear, denial and stigma and that they are bothered by the uncertainty of their stay at the hospital (Sun et al., 2020). Experiences of patients admitted into intensive care with a diagnosis of COVID-19 are reported to be manifested in emotions of fear, denial, anxiety and thoughts of impending death (Sahoo et al., 2020). This situation can also occur in all intensive care patients (Banzett et al., 2020). However, the sudden emergence of the COVID-19 pandemic, the lack of a known treatment, further increases the fear of death. The fear of death in ICU during the COVID-19 pandemic is much more higher than before the COVID-19 pandemic (Hao et al., 2020).

In this study, the patients revealed that they experienced difficulties in adapting to everyday life when they left the intensive care unit. Intensive care is of vital importance in reducing mortality rates among patients with COVID-19 (Xu et al., 2020). However, survivors of the illness are confronted with adversities following their intensive care and their treatment in the hospital wards, finding it hard to return to their daily activities and to continue with their customary routines (Batty et al., 2020; Hosey & Needham, 2020; Simpson & Robinson, 2020). COVID-19 patients have different kinds of negative experiences in the short and long term following their discharge from the hospital and depending upon the length of their stay (Batty et al., 2020; Hosey & Needham, 2020; Kang et al., 2020; Korupolu et al., 2020). It is stressed that after being discharged from the intensive care unit, 80% of COVID-19 patients experience physical, cognitive and/or psychological health issues that are generally identified as post-intensive care syndrome (Arnold et al., 2020). Patients who are discharged from the intensive care unit during the COVID-19 pandemic have the risk of contamination again outside. Therefore, patients are afraid of going back to intensive care and experiencing similar symptoms. The fear and weakness of returning to their daily activities are the different experiences in COVID-19 pandemic from discharge in ICU compared to the pandemic before the pandemic (Sun et al., 2020).

The patients however remarked that despite the adversities they faced during the COVID-19 infection following treatment, they were very much aware of the significance of their survival and return to health. They said they were more meticulous about following isolation rules and paid more attention to what they were eating. The uncertain duration of the illness, the high morality rates and the difficult intensive care conditions are stringent enough to make COVID-19 patients want to take care of their health to a greater degree (Sun et al., 2020).

This study showed that the patients’ experiences with healthcare professionals led them to acknowledge their dedicated and intensive efforts. The patients indicated that the healthcare team cared for them with devotion, maintaining constant contact at all times. The survivors of the COVID-19 infection also confided that their regard for doctors and other healthcare personnel had grown after this experience (Sahoo et al., 2020). The testimonies of patients reported in other studies show a respect for how the healthcare team met their physiological and psychological needs and thankfulness for their high sensitivity in providing care (Hao et al., 2020; Luo et al., 2020; Sun et al., 2020). Extraordinary sacrifices made by health professionals during the COVID-19 pandemic, despite the risks they face, has been acknowledged with admiration by the entire world (Huang et al., 2020; Kackin et al., 2020; Newby et al., 2020; World Health Organization, 2020a).

Most of the patients in the study said they looked at life differently now. Although there is a significant decline in the quality of life of patients after recovering from COVID- COVID-19 patients feel that they have matured and that their approach to life has changed (Brooks et al., 2020; Hosey & Needham, 2020; Sahoo et al., 2020). In a study that explored the perspective of patients on life after the COVID-19 infection, these survivors adopted a more positive perspective on life, realizing that health was even more important than money (Sahoo et al., 2020). It was underlined in another study that individuals turned toward their faith and made an effort to develop positive relations with the people around them (Brooks et al., 2020).

## Limitations

The only limitation of our study was that the data were collected through telephone calls. The opportunity to fully understand the emotions of our participants was very much restricted compared to face-to-face interviews because of the necessity to engage in a telephone conversation. In future studies, authors can use any video app for interviews, rather than just the phone.

## Conclusion

COVID-19 patients live with a fear of death caused by the uncertainty of their intensive care experience and the progression of the disease. COVID-19 may have both physical and psychosocial adverse effects on patients admitted to the ICU. The effects of the infectious disease take hold not only during their stay in the ICU but even after discharge. Nurses play a significant role in providing effective patient care during patients’ stay in the unit and in ensuring that they leave the intensive care unit with a positive outlook.

Care and communication with COVID-19 patients can be limited due to the rules of isolation. The first step in carrying out interventions in the ICU is to ensure that continuous communication with patients is maintained so that their orientation to the new circumstances can be achieved. The opportunity to have home care nursing should be provided after discharge from the hospital, patients should be followed up, and holistic care should be offered. Nursing interventions to patients missing their families can have compensated for the loss of family support and care during their critical illness.
